# Everyday discrimination, co-ethnic social support and mood changes in young adult immigrants in Germany–Evidence from an ecological momentary assessment study

**DOI:** 10.1016/j.jmh.2024.100212

**Published:** 2024-01-04

**Authors:** Heike Krüger

**Affiliations:** a Institute of Sociology and Social Psychology, University of Cologne, Albertus-Magnus-Platz, Köln 50923, Germany; b Institute of Sociology, RWTH Aachen University, Eilfschornsteinstraße 7, Aachen 52056, Germany

**Keywords:** Mood, Immigrant health, Discrimination, Social support, Intergroup contact, Ecological momentary assessment

## Abstract

**Objective:**

In the context of international migration flows, Germany is the second largest receiving country of migrants in the world. The aim of this study is to investigate the momentary mood effects of discrimination experiences for young adult immigrants and which social resources can buffer negative effects. A distinction is made between the importance of inter and intra-ethnic interaction partners in processing stressors.

**Method:**

Using an ecological momentary assessment design, first-generation migrants in Germany who had recently migrated from Poland, Turkey or Syria were interviewed three times a day over seven days in June 2021 (N individuals = 976; N observations = 11,470). The timing of the short surveys was chosen at random using a signal contingent sampling method. Participants reported their momentary mood and instances of discrimination, along with information on social support perception and interaction partners during the preceding hour in the context of their everyday lives. Hybrid mixed-effects regression models were estimated and the research questions were tested with three-way interactions.

**Results:**

The results indicate that perceived social support only moderately buffers the negative effect of everyday discrimination experiences on mood. A positive main effect on mood is observed for situational variations in perceived social support as well as for support from interaction partners.

**Conclusion:**

The findings illustrate that being embedded in supportive relationships is important in everyday life, regardless of the occurrence of stressors. Furthermore, the study suggests that the level of perceived support is more important for first-generation migrants than the ethnic origin of the support provider.

## Highlights

• Ecological momentary assessment study of the stress-buffering tendencies of inter- and intra-ethnic support for first-generation migrants in Germany.
• Hybrid mixed models reveal a significant association between perceived social support and mood, while support buffers the negative effect of discrimination only to a minor extent.
• The difference between intra- and inter-ethnic support is quite small, suggesting that the level of perceived social support is much more important for mood than the ethnic origin of those providing support.

## Introduction

A widely replicated finding in the context of migration research is the Healthy Immigrant Effect (HIE), whereby migrants have significantly better health outcomes compared to the host society (Kennedy et al., 2015). This difference is attributed to selection mechanisms and usually persists for years after migration, until there is an alignment with the host society in terms of health (Constant et al., 2018).

However, while this line of research tends to focus on physical health, research in the area of mental wellbeing points in the opposite direction. For example, first-generation migrants appear to have a higher prevalence of depression, post-traumatic stress disorder and anxiety disorders (Bas-Sarmiento et al., 2017; Close et al., 2016; Lee, 2019). Particularly for those with war-related refugee experiences, the effects of war trauma are of long-term relevance (Knaevelsrud et al., 2017; Długosz, 2023). First-generation migrants often face an increased number of stressors even after arrival in the host society: Distance from social contacts in the home country, social isolation in the host country, and legal problems. First-generation migrants are also particularly vulnerable to experiences of discrimination, which can further negatively affect mental health (Pearce et al., 2019), sleep quality (Alcántara et al., 2017), and cardiovascular health (Panza et al., 2019). In particular, these trends have also been confirmed for experiences of ethnic discrimination (e.g. Paradies et al., 2015; Stopforth et al., 2022).

### Perceived discrimination as a stressor

Stress is not a phenomenon that occurs only in connection with specific life events or catastrophes, but is an element of everyday life: “it typically concerns thoroughly socialized people engaged in the ordinary pursuits of life and driven by widely shared values and commitments.” (Pearlin et al., 1999: p. 396). Although stress is a part of every individual's life, the frequency, severity, and nature of the stressors to which individuals are exposed vary between social categories (Turner et al., 1995). First generation migrants in particular, are at increased risk of experiencing discrimination in the host society.

Experiences of discrimination represent an identity threat because they reveal a conflict between the social context and the individual's social identity. The perception of this conflict can lead to a lack of belonging on the one hand, and to feelings of exclusion and rejection on the other (Slepian and Jacoby-Senghor, 2020; Brance et al., 2022). In particular, experiences of social exclusion tend to be associated with negative emotions (Slepian and Jacoby-Senghor, 2020; MacDonald and Leary, 2005). Empirical evidence further suggests that experiences of discrimination promote the development of negative relational schemas. A survey of US adults identified concerns about rejection and invalidation as driving dimensions of social cognition that act as mediators between discrimination and depression (Mikrut et al., 2022).

For refugees, subjective social status in the host society is a key predictor of mental health (Correa-Velez et al., 2010). However, discrimination can also cause stress indirectly, for example when structural disadvantages lead to economic restrictions (Krieger, 2001), and poorer working conditions (Devkota et al., 2021). Experimental studies show that experiences of discrimination also operate through neuroendocrine pathways, as they are associated with increased cortisol levels (Korous et al., 2017). Ecological momentary assessment data provide evidence that discrimination experiences continue to influence cortisol levels the next day (Nam et al., 2022). Discrimination not only has an immediate impact on individuals, but also affects long-term health. Meta-analyses and systematic reviews show a negative association with self-reported health (Wanner and Pecoraro, 2023), and overall mental health (Cave et al., 2020). Against this background, it is important to explore which social resources can buffer the negative impact of discrimination on the mental health of first-generation migrants.

### Perceived discrimination and the role of social support

According to Lazarus and Folkman's (Lazarus and Folkman, 1984) transactional model of stress and coping, coping refers to behavioral patterns aimed at managing stressors and their consequences, either cognitively or through action. Coping can thus mitigate short-term effects on mood and long-term effects on mental and physical health. Social support is an important resource within the coping process (Thoits, 1995). The Buffering Hypothesis (Cohen and Wills, 1985) also emphasises the role of social support. In acute stress situations, such as experienced discrimination, social relationships can buffer the impact on health. For example, a functioning support system may help to resolve the underlying problem that triggers a stress response, or it may help with emotional coping by providing emotional support. These mechanisms lead to greater resilience in stressful situations, reducing vulnerability to mental illness. However, Cohen and Wills (1985) also assume that perceived social support can prevent a stressful event form causing a stress appraisal response in the first place. Thus, being embedded in a supportive network and being aware of the availability of instrumental and emotional support may reduce the likelihood of stressors being categorised as such, as their potential risk is perceived to be lower. An extensive number of studies have tested the general assumptions about the direct and buffering effects of social support on mental health. Results of meta-analyses are largerly in accordance (e.g. Rueger et al., 2016; Harandi et al., 2017). Buffering tendencies have also been shown in the context of discrimination, where social support was able to reduce the negative impact (Chou, 2012; Ajrouch et al., 2010).

The distinction between inter-ethnic and intra-ethnic relationships is highlighted in research on immigrant incorporation (e.g. Baerveldt et al., 2004; Portes and Sensenbrenner, 1993). In particular, first-generation migrants are often still closely connected to family members and friends from their home country. At the same time, the development of close relationships in the host society is complicated by language barriers and acculturation processes (Jasinskaja-Lahti et al., 2006). The first social contacts in the country of arrival are often members of the same ethnic group and are therefore more likely to be providers of social support in the first years after arrival (van Tubergen, 2015). In the case of racial discrimination, intra-ethnic interaction partners can strengthen in-group identification and thus mitigate the emerging sense of lack of belonging by making the ethnic group membership more salient and "in that they can bring information, help, share emotions and experiences about painful situations, or to enact specific strategies" (Berjot and Gillet, 2011: p. 6). In general, in-group ties seem to be negatively associated with the use of ruminative coping and may therefore be particularly effective in coping with stress (Ysseldyk et al., 2018). In contrast, it is to be expected that perceived support from members of the host society should attenuate feelings of rejection associated with experiences of discrimination, by counteracting the perceived exclusion through positive interactions, thereby buffering the negative effect on mood. This has been confirmed in relation to first-generation migrants in Italy, where feelings of social exclusion were mitigated mainly by social contacts with members of the host society (Marinucci et al., 2022).

Previous studies that have differentiated between the importance of inter- and intra-ethnic support in dealing with experiences of discrimination have yielded mixed results. For most of the studied migrant groups, intra-ethnic support had a positive effect and was able to mitigate the negative impact of discrimination experiences on mental health (Noh and Kaspar, 2003; Mossakowski and Zhang, 2014; Kim and Noh, 2016), with the exception of Vietnamese migrants in the Canadian context (Kim and Noh, 2016). However, both reinforcing and mitigating effects are found for interethnic support from members of the host society, depending on the migrant group (Jasinskaja-Lahti et al., 2006; Kim and Noh, 2016). A survey of refugees in Switzerland also indicates that intergroup friendships can mitigate the negative impact of post-migration living difficulties in general on mental health. However, the association between trauma and psychological distress was stronger for refugees with Swiss friends (Pouraghajan et al., 2023).

In part, these differences may be due to differences in the host societies and immigrant groups under study. In addition, previous studies have only used cross-sectional data. Thus, they have only been able to examine between-subject differences in aggregate perceptions of support and discrimination.

### Contribution

This study contributes to the discussion by using survey data from first-generation migrants in Germany who have recently migrated from Poland, Turkey or Syria. Using a smartphone-based experience sampling design, the study examines the momentary impact of discrimination experiences on mood changes. The study provides evidence on whether perceived social support at the situational level can mitigate the negative impact of discrimination. By collecting information on recent social interaction partners, it is possible to test whether individuals are less likely to report declining mood scores after a discrimination experience if they interact with an inter- or intra-ethnic support provider.

The current study makes three contributions to this line of research. First, it is based on a research design that allows for the distinction between within-person and between-person sources of variation. Thus, compared to previous studies, it adds the possibility of examining within-person variation in perceived discrimination, social support and mood. Substantial disparities between within and between effects would further underscore the need for researchers in the field to adopt similar designs in future studies. Second, the ecological momentary assessment method allows the measurement of the immediate consequences of discrimination experiences and their association with situational variation in support resources and partners. Measuring mechanisms at the situational level greatly reduces the influence of recall bias, in contrast to the retrospective self-reports of standard surveys (Schwarz, 2007). Collecting data in the context of regular day-to-day routines also increases ecological validity (Shiffman et al., 2008). Moreover, the research questions is tested on a relatively large sample of first-generation migrants in Germany, which is a sub-sample of a larger random sample. So far, the importance of inter- and intra-ethnic support has not been investigated in Germany. However, in the context of international migration flows, Germany is the second largest receiving country of migrants in the world (Germany, 1995; Nations, 2020).

Finally, the current study offers practical insights extending beyond academic discourse. The study's focus on first-generation immigrants in Germany from Poland, Turkey, or Syria offers valuable insights to practitioners working with these populations. It underscores the need to understand and address discrimination faced by these groups and how it affects their mental health. Examining inter- and intra-ethnic support as possible mitigating factors for the adverse effects of discrimination may aid in creating tailored support programms that cater to the distinctive social needs present in each migrant group.

## Methods

### Study design and participants

I use data from from the research project “Social integration and everyday life in Germany” (SOCIALBOND). A smartphone-based experience sampling study was conducted with a subsample of a large-scale two-wave panel study of young adult first-generation migrants in Germany. Participants in this panel study were identified using a random sample of registry data from each of the five cities with the most migrants from the respective group in Germany (ENTRA survey, Kristen and Seuring, 2021). In the second wave, all participants of Turkish, Syrian, or Polish origin had the opportunity to indicate whether they were interested in the smartphone-based study. 1078 individuals agreed to participate and 977 responded to at least one signal. The questionnaire was translated into Turkish, Arabic and Polish, following the TRAPD translation model (Harkness, 2003; Mohler et al., 2016).

The smartphone-based survey was an ecological momentary assessment study. After a pre-survey, participants were sent three short questionnaires every day (signals) for seven consecutive days in May 2021. In total, 21 signals were sent. The survey invitations were sent via SMS, and an existing internet connection was required to complete the short questionnaires. The first daily questionnaire was sent between 10 am and 1 pm, the second in the afternoon between 2 pm and 5 pm, and the third in the evening between 6 pm and 9 pm. The exact time of the survey invitation within these intervals was randomised. Participants had one hour to complete the short surveys, after which the survey link expired. 11,470 responses were received. The overall response rate was 55.96 %. The response rate differs between individuals of Turkish (57.94 %), Syrian (48.80 %) and Polish (60.14 %) origin. After listwise deletion, the analysis sample consists of 9282 signals, of which 2614 are from Syrians, 3753 from Poles and 2915 from Turks.

### Measures

*Discrimination:* Discrimination was measured using an adapted version of the Everyday Discrimination Scale (Williams et al., 1997). For seven different types of discrimination, respondents were asked whether they had experienced each type in the last hour (e.g. you were treated with less courtesy than other people; Cronbach's alpha 0.797). For the purposes of the analyses below, there is only a distinction between whether the respondent had experienced at least one of the seven types of discrimination in the last hour, and whether the respondent had not experienced any of the seven types of discrimination in the last hour.

*Perceived social support*: Perceived social support was measured with four items. Two items each measured the emotional (e.g. I am shown empathy for my situation) and practical (e.g. I feel supported in everyday life) dimensions of support. Respondents were asked to answer the four items using the temporal reference 'at the moment'. All four items were collapsed into a mean score index (Cronbach's alpha 0.886).

*Social support within interactions*: Each short questionnaire assessed whether respondents had interacted with other people in the past hour - examples of interactions were a (short) conversation, a purchase or an online communication. If an interaction had taken place, detailed follow-up questions were asked about one interaction partner selected by the respondent. For this interaction partner, the respondent was asked: "How strongly do you feel supported by the other person?”. The response options ranged from "0 not at all" to "4 very much". In addition, the perceived ethnic background of the interaction partner was recorded. This information was dichotomised, with 0 indicating partners of a different ethnicity and 1 indicating persons of the same ethnicity.

*Mood*: Mood was captured by a series of mood adjectives, as is common in ecological momentary assessment studies (de Vries et al., 2021). For each adjective, respondents rated the extent to which it described their feelings at the moment. The response options ranged from "0 not at all" to "4 very much". The adjectives relaxed, happy, enthusiastic and optimistic represent the dimension of positive mood (Cronbach's alpha 0.782). Negative mood was captured by the adjectives nervous, sad, downhearted, and angry (Cronbach's alpha 0.841). The mood scale was obtained by calculating a mean index over all eight items (Cronbach's alpha 0.846), the negative mood adjectives were reversed beforehand.

*Confounding variables*: Seven variables were considered as time-constant confounders. The first seven originate from the first wave of Entra. Thus, the data were collected about 2 years prior to this study. Gender was included because women tend to report poorer mental health (Campbell et al., 2021) and because social support resources also differ between the sexes (Flaherty and Richman, 1989). A distinction was made between female, male and diverse. Age at the time of the survey was entered as a continuous variable. Previous research suggests an association with both social support and mental health (Bell et al., 2019; Wrzus et al., 2013). I distinguished the three migrant groups in terms of country of origin as Syrians, Turks and Poles. Length of stay in Germany was measured in years, and language proficiency was self-reported. Migration-related indicators were classified as confounders because migration history has an impact on integration into the host society and thus on social resources (Oppedal et al., 2004). In addition, it is a factor related to health (Koneru et al., 2007; Juárez et al., 2018). Socio-economic indicators are the number of years spent in the education system and net household income. These were included because social support resources show a socio-economic gradient (Schafer and Vargas, 2016) and previous research also suggests a relationship with mood (Hao and Farah, 2020). In addition, global mental health was considered a confounder because of its importance as a predictor of situational mood (Armey et al., 2015) and because it shapes how we view social situations (Leskelä et al., 2008). The WHO-5 Well-Being Index (WHO, 1998), which was collected during the pre-survey, was used.

Two time-varying confounders were included: Time of day and day of week. Due to the survey design, time of day was included as a categorical variable rather than a continuous variable. A distinction was made between surveys in the morning, afternoon, and evening. These are confounding factors in that mood varies with time of day and day of week (Egloff et al., 1995; Stone et al., 2012). Furthermore, interaction partners typically differ across these temporal dimensions.

### Analytical strategy

To address the research question, a series of hybrid mixed-effects regression models (MRMs) (Hedeker and Gibbons, 2006) were estimated. Intercepts were allowed to vary between individuals ($\upsilon_{0i}$). Effects were decomposed into within-subject and between-subject effects. The within-subject effect was obtained by mean-centering: the subject-level mean is subtracted from each time-varying observation, thus measuring the deviation from the subject-level mean $\left({\text{X}}_{\text{ij}}-{\bar{X}}_{i}\right)$. The between-subject effect was identified by separately including the individual-level mean $\left({\bar{X}}_{i}=\sum_{j=1}^{n_{i}}{\text{X}}_{\text{ij}/}{\text{n}}_{\text{i}}\right)$ (Begg and Parides, 2003):

(1) $$\begin{array}{c} {\text{y}}_{\text{ij}}=\beta_{0}+\beta_{1}\left({\text{X}}_{\text{ij}}-{\bar{X}}_{i}\right)+\beta_{2}{\bar{X}}_{i}+\upsilon_{0i}+\varepsilon_{\text{ij}} \end{array}$$

To test the hypothesized buffering effects of social support, multiplicative interaction terms are included in the hybrid mixed-effects regression models. The interaction terms were decomposed into within-subject and between-subject effects using the same approach. First, the interaction term (${\text{X}}_{\text{ij}}{\text{Z}}_{\text{ij}}$) was generated, second the subject level mean of the interaction term across all observations was generated ($\bar{X_{i}Z_{i}}$), third to generate the within subject level term the interaction term was cluster mean centered by subtracted the subject level mean from each observation $\left({\text{X}}_{\text{ij}}{\text{Z}}_{\text{ij}}-\bar{X_{i}Z_{i}}\right)$(Schunck, 2013). This results in the following equation for testing the two-way interaction:

(2) $$\begin{array}{c} {\text{y}}_{\text{ij}}=\beta_{0}+\beta_{1}\left({\text{X}}_{\text{ij}}-{\bar{X}}_{i}\right)+\beta_{2}{\bar{X}}_{i}+\beta_{3}\left({\text{Z}}_{\text{ij}}-{\bar{Z}}_{i}\right)+\beta_{4}{\bar{Z}}_{i}+\beta_{5}\left({\text{X}}_{\text{ij}}{\text{Z}}_{\text{ij}}-\bar{X_{i}Z_{i}}\right)+\beta_{6}\left(\bar{X_{i}Z_{i}}\right)+\upsilon_{0i}+\varepsilon_{\text{ij}} \end{array}$$

The between-subject effects indicate the averaged association between the independent variables and the dependent variable across all observations: e.g. the extent to which an individual's average perceived social support is associated with an individual's average mood level. Whereas within-subject effects indicate the extent to which changes in the explanatory variable within the same individual are related to changes in the individual's outcome variable: e.g. how changes in the individual's perception of social support across observations are related to situational variations in the individual's mood.

Standard random effects models do not distinguish between and within estimators, implicitly assuming that they are identical. However, in cases where they differ, the resulting average of these two effects can be difficult to interpret. In contrast, hybrid mixed models combine the advantages of between-effects and fixed-effects regressions (Bell et al., 2019). The demeaning of the variables ensures that omitted variables at the individual level (between effects) cannot introduce bias into the within estimators. However, using pure fixed effects models would have the consequence that the "… de-meaned FE specification reveals almost nothing about the level-2 entities in the model" (Bell et al., 2019: p. 1058).

Missing values on variables relevant to the analyses were treated with listwise deletion. The proportion of missing values is 0.19. No restrictions were applied with respect to completeness across all observations. Therefore, for each individual, all time points with complete information on all relevant variables were included.

## Results

### Descriptive statistics

Table 1 presents descriptive statistics at the signal level from the experience sampling data and at the respondent level from the first ENTRA wave and the pre-survey. In the overall sample, the gender ratio is balanced. However, there is a higher proportion of men among Syrian respondents (69.7 %) and a higher proportion of women among Polish respondents (70.5 %). The average age is around 29 years. At the time of the first ENTRA wave, the Syrian participants had been in Germany for an average of 3.28 years, the Turkish participants for 1.87 years, and the Polish participants for about 2 years. In terms of the WHO-5 Well-Being Index, the Syrian respondents have the highest mean score (*M* = 14.29), in contrast to the Turkish (*M* = 13.33; Tukey post-hoc test for comparison of means, −0.967 +/- 0.465, *p* = .095) and Polish (*M* = 13.21; Tukey post-hoc test for comparison of means, −1.081 +/- 0.443, *p* = .040) respondents.

**Table 1:** Descriptive statistics analysis sample.

	Full sample		Syrian		Polish		Turkish	
Variable	Mean	SD	Mean	SD	Mean	SD	Mean	SD
Experience sampling data								
Mood	3.795	.718	3.767	.723	3.906	.725	3.677	.683
Discrimination (last hour)	.062	.241	.085	.279	.045	.207	.063	.244
Perceived support	3.703	.911	3.606	.821	3.828	.95	3.631	.919
Same-ethnic interaction partner *	.506	.5	.619	.486	.476	.5	.477	.5
Supportive interaction*	3.836	1.213	3.75	1.167	3.787	1.256	3.967	1.164
Time	.	.	.	.	.	.	.	.
Morning	.335	.472	.333	.472	.342	.475	.327	.469
Afternoon	.333	.471	.342	.475	.326	.469	.335	.472
Evening	.332	.471	.324	.468	.332	.471	.338	.473
Weekday	.	.						
Mon	.175	.38	.178	.382	.172	.377	.175	.380
Tue	.16	.367	.168	.374	.158	.365	.156	.363
Wed	.149	.356	.15	.357	.149	.356	.15	.357
Thu	.14	.347	.141	.348	.139	.346	.141	.348
Fri	.128	.334	.124	.33	.129	.336	.129	.335
Sat	.124	.329	.12	.325	.128	.334	.123	.328
Sun	.125	.33	.121	.326	.126	.332	.127	.333
N (signals)	9282		2614		3753		2915	
Pre-survey/wave 1 ENTRA								
Gender	.	.	.	.	.	.	.	.
Divers	.003	.052	.	.	.004	.060	.004	.066
Male	.489	.5	.697	.461	.292	.455	.520	.501
Female	.509	.5	.303	.461	.705	.457	.476	.501
Age	29.192	5.211	28.823	5.085	29.637	4.913	29.017	5.657
Years of education	15.871	3.315	15.121	3.335	16.47	2.736	15.893	3.774
Income	4.128	2.942	2.061	1.568	5.577	2.791	4.437	3.005
Length of stay (years)	2.356	1.237	3.281	.97	1.996	1.073	1.865	1.155
Language proficiency	3.551	1.171	3.104	1.02	3.687	1.254	3.834	1.08
Well-Being	13.586	5.006	14.294	5.136	13.214	4.844	13.328	5.015
N (individuals)	741		231		281		229	

⁎ Mean and SD refer only to those signals with reported interaction (N full sample = 4797, N Syrians = 975, N Pols = 2320, N Turks = 1502).

For the EMA measures, slight mean differences are observed for mood. Polish respondents have a slightly higher mean (*M* = 3.91), compared to Syrian (*M* = 3.77; Tukey post-hoc test for comparison of means, 0.140 +/- 0.018, *p* = .000) and Turkish respondents (*M* = 3.68; Tukey post-hoc test for comparison of means, −0.229 +/- 0.018, *p* = .000). Syrian respondents reported experiencing discrimination in 8.5 % of signals, Turkish respondents in 6.3 % (Tukey post-hoc test for comparison of means with Syrians, −0.022 +/- 0.007, *p* = .003) and Polish respondents in 4.5 % of signals (Tukey post-hoc test for comparison of means with Syrians, −0.040 +/- 0.006,*p* = .000). On average, Polish respondents reported more perceived social support (*M* = 3.83) than Turkish (*M* = 3.63; Tukey post-hoc test for comparison of means, −0.197 +/- 0.022, *p* = .000) and Syrian (*M* = 3.61; Tukey post-hoc test for comparison of means, 0.222 +/- 0.023, *p* = .000) respondents. Considering only those signals in which an interaction took place in the last hour, on average, the Turkish respondents found their interaction partner the most supportive (*M* = 3.97), compared to the Polish (*M* = 3.79; Tukey post-hoc test for comparison of means, 0.180 +/- 0.040, *p* = .000) and Syrian (*M* = 3.75; Tukey post-hoc test for comparison of means, 0.217 +/- 0.050, *p* = .000) respondents. When an interaction occurred, it was with an interaction partner of the same ethnicity in 61.9 % of signals for the Syrians, in 47.7 % of signals for the Turks and in 47.6 % of signals for the Poles.

### Hybrid mixed models

#### Recent discrimination and situational perceived social support

The hypothesised role of perceived social support in mitigating the negative impact of perceived discrimination experiences is tested through interaction effects. Table 2 presents results from hybrid mixed-effects regression models, including an interaction between experiences of discrimination within the last hour and perceived situational social support. (see online appendix for main effect models). The results in Model 1 are based on the full sample, while the results for Syrian respondents are presented in Model 2, for Polish respondents in Model 3, and for Turkish respondents in Model 4.

**Table 2:** Hybrid mixed-effects linear regression, two-way interaction effects perceived social support and discrimination, full sample (all signals).

	Full sample (1)	Syrians (2)	Poles (3)	Turks (4)
	Mood a	Mood b	Mood b	Mood b
Between subject effects:				
Discrimination	−0.545*	−0.449	−1.446⁎⁎⁎	.389
	(0.292)	(0.553)	(0.523)	(0.469)
Perceived support	.317⁎⁎⁎	.319⁎⁎⁎	.294⁎⁎⁎	.37⁎⁎⁎
	(0.021)	(0.044)	(0.033)	(0.035)
Discrimination*support	.059	.026	.263	−0.172
	(0.089)	(0.159)	(0.178)	(0.145)
Within subject effects:				
Discrimination	−0.512⁎⁎⁎	−0.305⁎⁎	−0.598⁎⁎⁎	−0.596⁎⁎⁎
	(0.076)	(0.145)	(0.128)	(0.127)
Perceived support	.437⁎⁎⁎	.442⁎⁎⁎	.439⁎⁎⁎	.424⁎⁎⁎
	(0.011)	(0.021)	(0.016)	(0.02)
Discrimination*support	.078⁎⁎⁎	.021	.096⁎⁎	.103⁎⁎⁎
	(0.023)	(0.044)	(0.04)	(0.039)
Constant	1.901⁎⁎⁎	1.883⁎⁎⁎	2.394⁎⁎⁎	1.54⁎⁎⁎
	(0.136)	(0.301)	(0.218)	(0.21)
var(_cons)	.120⁎⁎⁎	.123⁎⁎⁎	.120⁎⁎⁎	.103⁎⁎⁎
	(0.008)	(0.014)	(0.012)	(0.012)
var(Residual)	.184	.182⁎⁎⁎	.187⁎⁎⁎	.179⁎⁎⁎
	(0.003)	(0.005)	(0.005)	(0.005)
N (level 1)	9282	2614	3753	2915
N (level 2)	741	231	281	229

Standard errors are in parentheses

⁎⁎⁎ *p* < .01,.

⁎⁎ *p* < .05,.

⁎ *p* < .1.

ab random effects adjusted for time of day, weekday, gender, years of education, income, length of stay, language proficiency, well-being.

a random effects also adjusted for country of origin.

**Fig. 1:**
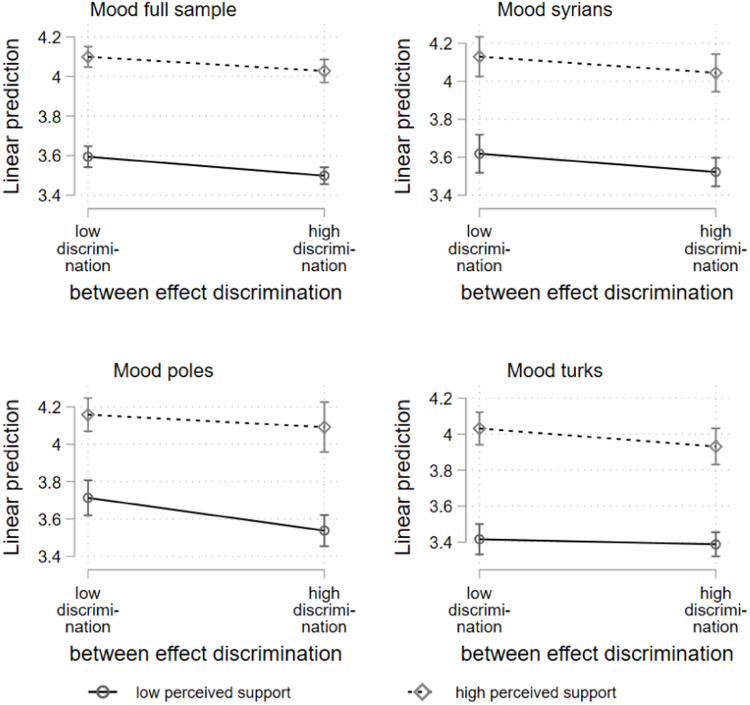
Hybrid mixed-effects linear regression, between subject effects; two-way interaction discrimination, and perceived social support (low = mean-1SD; high = mean+1SD).

With regard to the between subject effects, the results indicate no significant interaction between recent discrimination and perceived social support. As shown in Fig. 1, individuals who report many discrimination experiences across the signals tend to report lower mood scores than those individuals who report few discrimination experiences. With regard to social support, the graph suggests that individuals with on average high perceived social support across the signals tend to report higher mood values than individuals with on average low perceived social support. However, the mood difference between respondents with low or high occurrence of discrimination is not substantially different between individuals with a high mean of perceived social support. A slight buffering tendency is only visible for the Poles, for whom the mood difference between individuals with a lot and little discrimination is somewhat smaller for individuals with a high average social support perception (*p* = .139). A different trend is indicated for the Turks. Individuals with little and much experience of discrimination differ more strongly in their mood if they have a higher average support perception (*p* = .236).

**Fig. 2:**
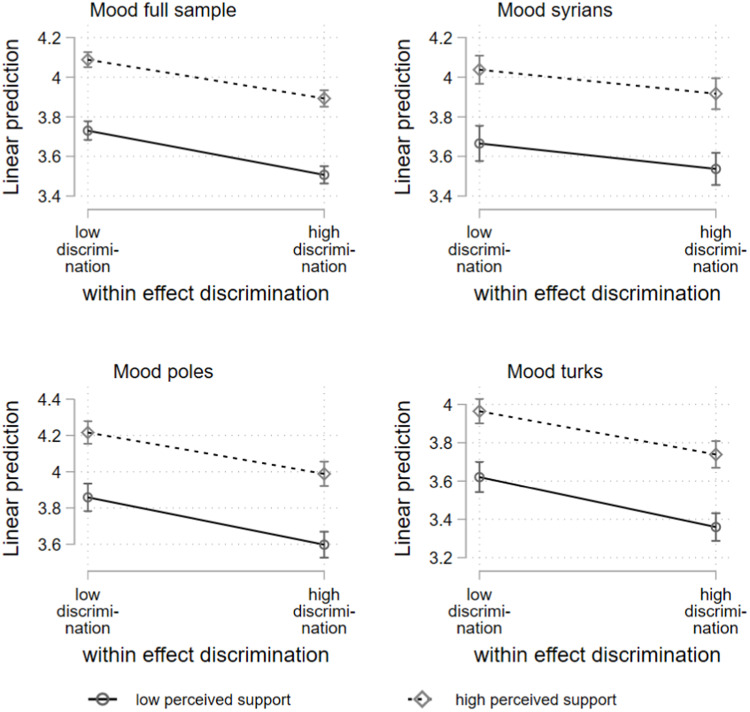
Hybrid mixed-effects linear regression, within subject effects; two-way interaction discrimination, and perceived social support (low= = mean-1SD; high = mean+1SD).

The effects at the within-subject level shown in Table 2 indicate a significant interaction effect for the full sample, as well as the Poles and Turks. Fig. 2 provides a graphical representation. At the Within Subject level, situations with a lot of discrimination are associated with worse mood than those with little discrimination. Moreover, within all levels of discrimination, mood is always better when situational perceived social support is high. The difference in mood between situations with a lot and little perceived support is larger in situations with discrimination. Thus, the decline in mood seems to be greater when there is low perceived social support compared to high perceived support availability.

However, the magnitude of this difference is relatively small. For the full sample the predicted margins show that the difference in mood between sampling moments with low (−1SD) and high (+1SD) perceptions of social support only increased by 0.03 scale points between situations with low (−1SD) and high (+1SD) discrimination.

#### Recent discrimination and situational intra- and inter-ethnic support

Testing the role of the ethnicity of the support provider for the hypothesised buffering effect of situational social support requires the estimation of three-way interactions. Table 3 presents the results of hybrid mixed-effects regression models depicting the multiplicative interaction between discrimination experiences within the last hour, situational social support from the interaction partner and the ethnicity of the support provider (see online appendix for main effect models). The analyses were calculated for the full sample (Model 1) as well as separately for the subsample of Syrians (Model 2), Poles (Model 3), and Turks (Model 4).

**Table 3:** Hybrid mixed-effects linear regression, three-way interaction effects support, inter/intra-ethnic contact and discrimination (time points with interactions only).

	Full sample (1)	Syrians (2)	Poles (3)	Turks (4)
	Mood a	Mood b	Mood b	Mood b
Between subject effect:				
Discrimination	−0.072	−0.709⁎⁎	.673	.174
	(0.198)	(0.297)	(0.485)	(0.348)
Support (interaction)	.198⁎⁎⁎	.024	.271⁎⁎⁎	.235⁎⁎⁎
	(0.029)	(0.061)	(0.046)	(0.046)
Same-ethnic interaction partner (interaction)	.146	−0.632*	1.098⁎⁎⁎	−0.465
	(0.198)	(0.353)	(0.317)	(0.344)
Discrimination*ethnicity	−0.119	−0.017	−1.17	1.275*
	(0.433)	(0.729)	(0.826)	(0.74)
Discrimination*support	−0.088*	.112	−0.513⁎⁎⁎	−0.169*
	(0.052)	(0.078)	(0.16)	(0.09)
Support*same ethnic	−0.048	.154*	−0.266⁎⁎⁎	.078
	(0.047)	(0.086)	(0.075)	(0.081)
Discrimination*support*same ethnic	−0.005	−0.008	.251	−0.296
	(0.119)	(0.189)	(0.275)	(0.202)
Within subject effect:				
Discrimination	−0.39⁎⁎⁎	−0.769⁎⁎⁎	−0.318⁎⁎	−0.339⁎⁎
	(0.084)	(0.182)	(0.14)	(0.133)
Support (interaction)	.062⁎⁎⁎	.033	.075⁎⁎⁎	.044⁎⁎
	(0.01)	(0.03)	(0.013)	(0.019)
Same-ethnic interaction partner (interaction)	−0.103*	−0.236*	−0.111	−0.026
	(0.061)	(0.138)	(0.085)	(0.121)
Discrimination*ethnicity	−0.07	.172	.235	−0.599*
	(0.164)	(0.304)	(0.256)	(0.315)
Discrimination*support	−0.023	.107*	−0.067	−0.032
	(0.027)	(0.058)	(0.047)	(0.042)
Support*same ethnic	.021	.053	.023	.004
	(0.015)	(0.037)	(0.021)	(0.029)
Discrimination*support*same ethnic	.056	−0.045	.009	.189⁎⁎
	(0.046)	(0.089)	(0.073)	(0.082)
Constant	2.377⁎⁎⁎	3.121⁎⁎⁎	2.53⁎⁎⁎	2.122⁎⁎⁎
	(0.171)	(0.368)	(0.265)	(0.255)
var(_cons)	.122⁎⁎⁎	.124⁎⁎⁎	.116⁎⁎⁎	.091⁎⁎⁎
	(0.009)	(0.019)	(0.013)	(0.014)
var(Residual)	.221⁎⁎⁎	.207⁎⁎⁎	.226⁎⁎⁎	.214⁎⁎⁎
	(0.005)	(0.011)	(0.007)	(0.009)
N (level 1)	4797	975	2320	1502
N (level 2)	702	210	273	219

Standard errors are in parentheses

⁎⁎⁎ *p* < .01,.

⁎⁎ *p* < .05,.

⁎ *p* < .1.

ab random effects adjusted for time of day, weekday, gender, years of education, income, length of stay, language proficiency, well-being.

a random effects also adjusted for country of origin.

**Fig. 3:**
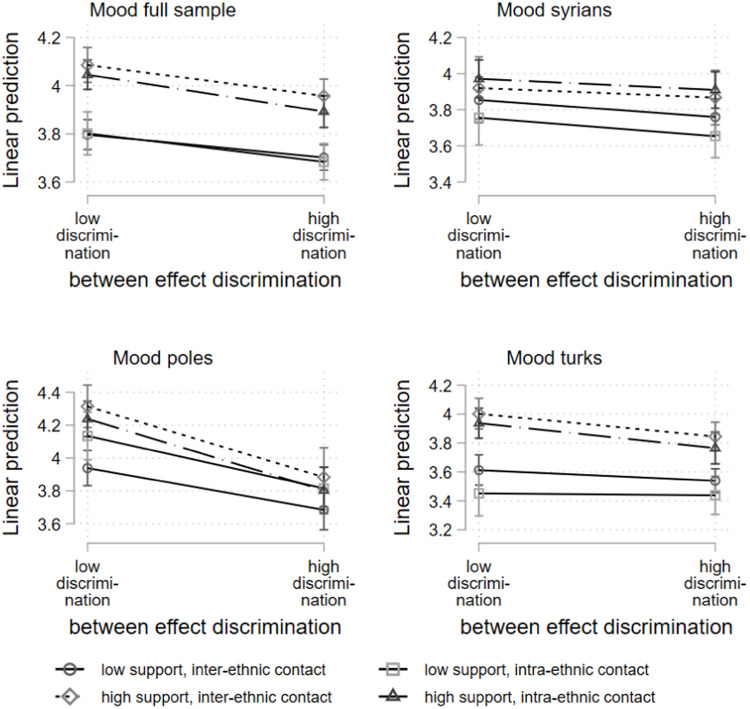
Hybrid mixed-effects linear regression, between subject effects; three-way interaction discrimination, support interaction partner, and ethnicitiy of interaction partner (low = mean-1SD; high = mean+1SD).

The results of the between-subjects effects from Table 3 are presented in Fig. 3. The main effect of social support is still evident when the ethnicity of the interaction partner is taken into account. Individuals who report high mean levels of support from their interaction partners are more likely to report higher mean mood scores. For Poles and Turks, individuals with more inter-ethnic contact tend to have higher mood scores; for Syrians, a high proportion of intra-ethnic contact is associated with better mood scores. For the full sample, as well as for the Poles and Turks, there is also a tendency for mood differences to be smaller between individuals with many reported experiences of discrimination than for individuals with many reported experiences of discrimination. However, compared to Fig. 1, the slopes show less pronounced trends with greater overlap of confidence intervals.

**Fig. 4:**
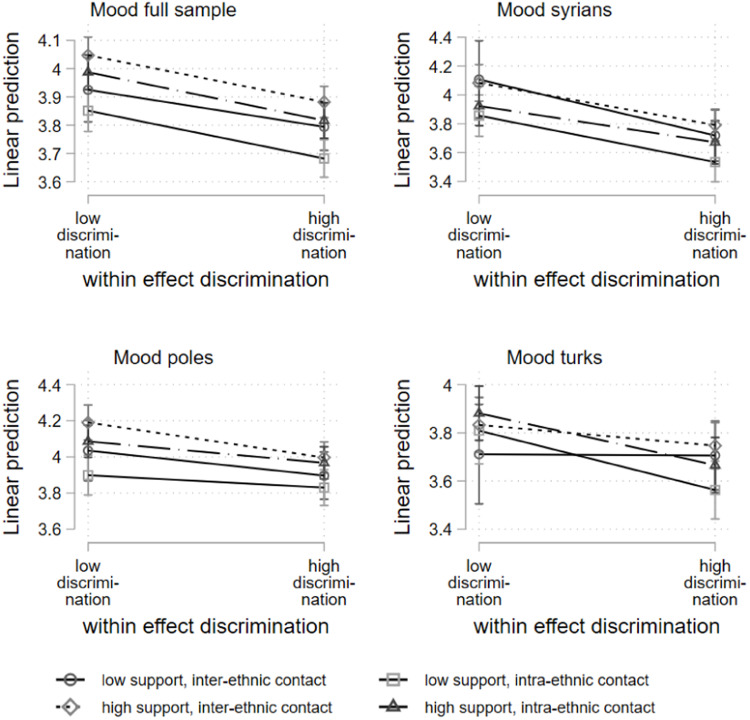
Hybrid mixed-effects linear regression, within subject effects; three-way interaction discrimination, support interaction partner, and ethnicitiy of interaction partner (low = mean-1SD; high = mean+1SD).

The effects at the within-subject level shown in Table 3 indicate partially significant interaction terms, but the graphical representation of the results in Fig. 4 illustrates that the confidence intervals strongly overlap. In addition, the slopes are relatively close to each other indicating only minor effect differences. Within the full sample and the three subgroup analyses, there is a negative relationship between situations with discrimination above the personal average and mood. Overall, there is no marked buffering effect of social support on the negative impact of discrimination experiences. However, there is evidence of a main effect of social support on mood. For this operationalisation, the results indicate that situations with high perceived support from the interaction partner are associated with better mood scores than situations in which the interaction partner is perceived as less supportive, irrespective of discrimination. In the full sample model, the highest mood scores are associated with support from an inter-ethnic interaction partner, although greater heterogeneity and overlap are evident in the subgroup analyses.

## Discussion

The aim of this study was to examine the extent to which perceived social support can mitigate the negative effects of discrimination among first-generation Syrian, Polish and Turkish immigrants in Germany. The study extends the existing literature by using smartphone-based experience data, which allows situational variation to be examined rather than aggregated information. In addition, the study investigated the importance of the ethnic background of the interaction partner.

The findings indicate that social support is an important resource for mood, independent of the occurrence of (perceived) discrimination. Thus, the results support the main effect model of social support. According to this model, the availability of social support has a positive effect on mental health even in the absence of stressor (Cohen and Wills, 1985; Thoits, 2011). The analyses also provide evidence for a buffering effect of the negative impact of discrimination by perceived social support, but the effect is of a minor magnitude.

Intra-ethnic support seems to be more relevant for Syrian respondents, while Poles and Turks tend to benefit more from inter-ethnic support in terms of mood, especially in contexts of high discrimination. However, the magnitude of the differences between intra-ethnic and inter-ethnic support is rather small in all models, suggesting that it is not so much ethnicity as the level of perceived social support that matters for mood. The findings here contrast with previous cross-sectional research suggesting differences in the importance of intra- and inter-ethnic support (Jasinskaja-Lahti et al., 2006; Mossakowski and Zhang, 2014; Kim and Noh, 2016; Juang et al., 2016). As the results of previous research suggest heterogeneity in importance across ethnic groups, they may be indicative of group differences in access to social resources. By using individual fixed effects, the present study should provide estimates that are less biased with respect to these issues.

In addition, situational changes in the perceived availability of social support appear to be more relevant to mood than perceived support from specific interaction partners. The findings are consistent with previous research suggesting that general perceptions of the availability of social support are a more consistent predictor of mental health than actual support received (Wills and Shinar, 2000).

### Limitations and future research

This study is subject to several limitations. First, the assumed direction of influence is not the only theoretically possible one. It is conceivable that lower mood may lead to a different evaluation of interactions, so that they are more likely to be classified as discriminatory or unsupportive. Previous research indicates that support is perceived to be less available and that the changed social behaviour of depressed individuals may lead to a loss of relationships and thus objectively reduce the available social support (Leskelä et al., 2008; Elmer and Stadtfeld, 2020). Therefore, it would be of interest for future research to determine which causal direction is of greater relevance.

Second, a more objective measure of available social support might help to address this causality issue, at least in part. However, previous research suggests that objective measures of social support have significantly less explanatory power for mental health than measures of perceived social support (Prati and Pietrantoni, 2010). The reason for this is that the level of support received tends to be higher when the need for support increases due to the acute onset of a stressor (Melrose et al., 2015) - in circumstances that are typically associated with poorer mental health. Future research using smartphone-based experience sampling approaches may provide new insights to better understand which objective indicators of social interactions and relationships are relevant to subjective perceptions of social support.

Third, the questionnaire only allowed participants to describe a maximum of one interaction partner. This restriction was made in order not to extend the length of the questionnaire too much and thus increase attrition between signals. It is quite possible that the question about the last interaction was related to the discrimination situation. Thus, perceptions of social support may be related to the interaction with the discriminator. However, this is likely to be a conservative bias that may lead to an underestimation of the effect.

Fourth, the distinction between inter- and intra-ethnic support is particularly important in the context of racial discrimination in order to counteract feelings of exclusion and rejection. Although participants in this study reported the perceived reasons for discrimination, it is not always possible to clearly identify whether these reasons were perceived as racially motivated. For future research, a larger number of observations with experiences of discrimination would be necessary for a detailed examination. It would also be important to determine whether ethnicity is a determining factor and to collect further information on the ethnic background of the discriminator.

Future research should also exploit the potential of ecological momentary assessment studies to gain further insight into other mechanisms that support first-generation immigrants' coping capabilities. Research based on annual longitudinal analyses indicates that positive affect may be important as an intermediate (Demirer et al., 2022).

## Conclusion

The results of this study once again highlight the negative consequences of discriminatory experiences. However, they also illustrate the importance of being embedded in a supportive network in everyday life, regardless of stressors. The study also suggests that the level of perceived support is more important for immigrants than the ethnic origin of the support provider.

Several policy implications can be derived from the findings of the present study. The detrimental effect of discrimination on mood emphasises the need for policymakers to implement and reinforce anti-discrimination measures in various social contexts, including workplaces, educational institutions, and public spaces, to create environments that facilitate positive mental health outcomes for immigrants. Thereby attention should be paid to the observation that the prevalence of discrimination differs considerably between ethnic groups. Under consideration of the varying importance of intra-ethnic and inter-ethnic support for different immigrant groups, tailored programmes should be developed to address the particular preferences and challenges faced by Syrian, Polish, and Turkish immigrants.

Facilitating social participation holds significant policy implications given that it is linked to an uplifting effect on mood that extends beyond sources of stress such as discrimination, as evidenced by the positive direct effect of social support. For social support to be accessible, individuals must first be incorporated into social structures. Encouraging integration into ethnically diverse social networks may also deter the creation of reinforced in-group and out-group barriers. Additionally, this study underlines the importance of promoting mental health literacy. It is crucial to educate first-generation immigrants about the affirmative influence of social support on mental health.

## CRediT authorship contribution statement

**Heike Krüger:** Conceptualization, Data curation, Formal analysis, Funding acquisition, Investigation, Methodology, Project administration, Resources, Software, Supervision, Validation, Visualization, Writing – original draft, Writing – review & editing.

## Declaration of competing interest

The authors declare the following financial interests/personal relationships which may be considered as potential competing interests:

This research was funded by the European Research Council (ERC) under the European Union's Horizon 2020 research and innovation programme (Grant agreement No. 716,461). Funded by the Deutsche Forschungsgemeinschaft (DFG, German Research Foundation) under Germany's Excellence Strategy—EXC 2126/1–390,838,866. I acknowledge support for the Article Processing Charge from the DFG (German Research Foundation, 491454339). The content is solely the responsibility of the author and does not necessarily represent the official views of the study sponsors.

## Appendix Supplementary materials

Image, application 1
