# Supplementary material for: Everyday discrimination, co-ethnic social support and mood changes in young adult immigrants in Germany–Evidence from an ecological momentary assessment study
Source: J Migr Health. 2024 Jan 4;9:100212. doi: 10.1016/j.jmh.2024.100212 (PMC10820660; doi:10.1016/j.jmh.2024.100212)
Supplement: Supplementary file 1 [file mmc1.docx]

# Supporting Information for review and online publication only

**Table A1: Matrix of correlations (analysis sample, all signals)**

| Variables | (1) | (2) | (3) | (4) | (5) | (6) | (7) | (8) | (9) | (10) | (11) |  |
| --- | --- | --- | --- | --- | --- | --- | --- | --- | --- | --- | --- | --- |
| (1) Mood | 1.00 |  |  |  |  |  |  |  |  |  |  |  |
| (2) Discrimination (last hour) | -0.22 | 1.00 |  |  |  |  |  |  |  |  |  |  |
| (3) Perceived support | 0.56 | -0.19 | 1.00 |  |  |  |  |  |  |  |  |  |
| (4) Time of day | 0.02 | -0.03 | 0.01 | 1.00 |  |  |  |  |  |  |  |  |
| (5) Weekday | 0.02 | -0.14 | -0.01 | 0.02 | 1.00 |  |  |  |  |  |  |  |
| (6) Age | 0.07 | -0.01 | 0.03 | -0.01 | 0.00 | 1.00 |  |  |  |  |  |  |
| (7) Years of education | 0.04 | -0.05 | 0.08 | 0.00 | 0.02 | 0.24 | 1.00 |  |  |  |  |  |
| (8) Income | 0.12 | -0.05 | 0.16 | -0.00 | 0.01 | 0.30 | 0.24 | 1.00 |  |  |  |  |
| (9) Language proficiency | -0.01 | -0.03 | -0.02 | -0.01 | 0.00 | 0.34 | 0.05 | 0.23 | 1.00 |  |  |  |
| (10) Length of stay (years) | 0.04 | 0.02 | -0.02 | 0.00 | -0.01 | 0.03 | -0.13 | -0.24 | -0.35 | 1.00 |  |  |
| (11) Well-Being | 0.42 | -0.07 | 0.35 | 0.00 | -0.01 | 0.08 | 0.06 | 0.00 | -0.08 | 0.04 | 1.00 |  |
|  | | | | | | | | | | | | |

**Table A2: Matrix of correlations (analysis sample, time points with interactions only)**

| Variables | (1) | (2) | (3) | (4) | (5) | (6) | (7) | (8) | (9) | (10) | (11) | (12) | (13) |  |
| --- | --- | --- | --- | --- | --- | --- | --- | --- | --- | --- | --- | --- | --- | --- |
| (1) Mood | 1.00 |  |  |  |  |  |  |  |  |  |  |  |  |  |
| (2) Discrimination (last hour) | -0.27 | 1.00 |  |  |  |  |  |  |  |  |  |  |  |  |
| (3) Perceived support | 0.55 | -0.24 | 1.00 |  |  |  |  |  |  |  |  |  |  |  |
| (4) Same ethnicity | 0.03 | -0.05 | -0.00 | 1.00 |  |  |  |  |  |  |  |  |  |  |
| (5) Supportive interaction | 0.28 | -0.19 | 0.38 | 0.25 | 1.00 |  |  |  |  |  |  |  |  |  |
| (6) Time of day | 0.04 | -0.03 | 0.02 | 0.09 | 0.03 | 1.00 |  |  |  |  |  |  |  |  |
| (7) Weekday | 0.08 | -0.12 | 0.04 | 0.08 | 0.04 | 0.06 | 1.00 |  |  |  |  |  |  |  |
| (8) Age | 0.05 | -0.01 | 0.00 | 0.04 | -0.00 | -0.00 | 0.04 | 1.00 |  |  |  |  |  |  |
| (9) Years of education | 0.03 | -0.05 | 0.06 | -0.05 | -0.01 | -0.00 | 0.05 | 0.28 | 1.00 |  |  |  |  |  |
| (10) Income | 0.09 | -0.07 | 0.14 | -0.06 | 0.07 | -0.01 | 0.06 | 0.31 | 0.24 | 1.00 |  |  |  |  |
| (11) Length of stay (years) | 0.02 | 0.05 | -0.02 | 0.01 | 0.00 | -0.01 | -0.03 | 0.04 | -0.12 | -0.21 | 1.00 |  |  |  |
| (12) Language proficiency | -0.04 | -0.04 | -0.06 | 0.12 | -0.00 | -0.01 | 0.03 | 0.32 | 0.08 | 0.18 | -0.28 | 1.00 |  |  |
| (13) Well-Being | 0.42 | -0.09 | 0.35 | -0.02 | 0.15 | -0.00 | 0.01 | 0.12 | 0.04 | 0.02 | 0.04 | -0.05 | 1.00 |  |
|  | | | | | | | | | | | | | | |

Tabelle A3: Between-cluster, and within-cluster variance, ICC

| Variable | Between-cluster variance | Within-cluster variance | ICC |
| --- | --- | --- | --- |
| Mood | .301 | .231 | .566 |
| Discrimination (last hour) | .019 | .047 | .291 |
| Perceived support | .642 | .197 | .765 |
| Same ethnicity | .082 | .168 | .330 |
| Supportive interaction | .392 | 1.09 | .264 |
| Intra-class correlation is defined as: Variance level 2 /( variance level 1 + variance level 2) Variance estimation based on empty mixed model | | | |

**Table A4: Multilevel mixed-effects linear regression, main effects (analysis sample, all signals)**

|  | Full sample  (1) | Syrians  (2) | Poles  (3) | Turks  (4) |  |
| --- | --- | --- | --- | --- | --- |
|  | Mood ^a^ | Mood ^b^ | Mood ^b^ | Mood ^b^ |  |
|  |  |  |  |  |  |
| Between subject effect: |  |  |  |  |  |
| Discrimination | -.357*** | -.36*** | -.725*** | -.145 |  |
|  | (.078) | (.113) | (.19) | (.129) |  |
| Perceived support | .322*** | .322*** | .307*** | .356*** |  |
|  | (.02) | (.04) | (.032) | (.033) |  |
|  |  |  |  |  |  |
| Within subject effect: |  |  |  |  |  |
| Discrimination | -.268*** | -.237*** | -.305*** | -.276*** |  |
|  | (.022) | (.038) | (.04) | (.039) |  |
| Perceived support | .445*** | .445*** | .447*** | .436*** |  |
|  | (.011) | (.02) | (.016) | (.019) |  |
|  |  |  |  |  |  |
| Constant | 1.886*** | 1.867*** | 2.363*** | 1.574*** |  |
|  | (.134) | (.284) | (.218) | (.209) |  |
| var(_cons) | .120*** | .123*** | .121*** | .103*** |  |
|  | (.008) | (.014) | (.012) | (.012) |  |
| var(Residual) | .184*** | .182*** | .187*** | .179*** |  |
|  | (.003) | (.005) | (.005) | (.005) |  |
| N (level 1) | 9282 | 2614 | 3753 | 2915 |  |
| N (level 2) | 741 | 231 | 281 | 229 |  |
| *Standard errors are in parentheses* | | | | | |
| **** p<.01, ** p<.05, * p<.1* | | | | | |
| ^a^ adjusted for time of day, weekday, gender, country of origin, years of education, income, length of stay, language proficiency, well-being (all random effects)  ^b^ adjusted for time of day, weekday, gender, years of education, income, length of stay, language proficiency, well-being (all random effects) | | | | | |

**Table A5: Multilevel mixed-effects linear regression, main effects (analysis sample, time points with interactions only)**

|  | Full sample  (1) | Syrians  (2) | Poles  (3) | Turks  (4) |  |
| --- | --- | --- | --- | --- | --- |
|  | Mood ^a^ | Mood ^b^ | Mood ^b^ | Mood ^b^ |  |
|  |  |  |  |  |  |
| Between subject effect: |  |  |  |  |  |
| Discrimination | -.447*** | -.331** | -1.055*** | -.315** |  |
|  | (.099) | (.149) | (.236) | (.152) |  |
| Support (interaction) | .168*** | .125*** | .152*** | .226*** |  |
|  | (.021) | (.04) | (.036) | (.034) |  |
| Ethnicity (interaction) | -.054 | -.047 | .019 | -.107 |  |
|  | (.049) | (.096) | (.083) | (.079) |  |
|  |  |  |  |  |  |
| Within subject effect: |  |  |  |  |  |
| Discrimination | -.412*** | -.414*** | -.427*** | -.403*** |  |
|  | (.031) | (.059) | (.051) | (.055) |  |
| Support (interaction) | .07*** | .076*** | .079*** | .048*** |  |
|  | (.008) | (.019) | (.01) | (.014) |  |
| Ethnicity (interaction) | -.018 | -.045 | -.009 | -.009 |  |
|  | (.019) | (.044) | (.027) | (.033) |  |
|  |  |  |  |  |  |
| Constant | 2.483*** | 2.724*** | 2.938*** | 2.124*** |  |
|  | (.154) | (.327) | (.248) | (.226) |  |
| var(_cons) | .124*** | .124*** | .128*** | 0.095*** |  |
|  | (.009) | (.019) | (.015) | (.014) |  |
| var(Residual) | .221*** | .210*** | .227*** | .216*** |  |
|  | (.005) | (.011) | (.007) | (.009) |  |
| N (level 1) | 4797 | 975 | 2320 | 1502 |  |
| N (level 2) | 702 | 210 | 273 | 219 |  |
| *Standard errors are in parentheses* | | | | | |
| **** p<.01, ** p<.05, * p<.1* | | | | | |
| ^a^ adjusted for time of day, weekday, gender, country of origin, years of education, income, length of stay, language proficiency, well-being (all random effects)  ^b^ adjusted for time of day, weekday, gender, years of education, income, length of stay, language proficiency, well-being (all random effects) | | | | | |
